# EDTA tubes are suitable for insulin and C-peptide measurement in resource-limited settings and can be stored at room temperature for up to 24 hours

**DOI:** 10.1371/journal.pone.0312065

**Published:** 2025-06-30

**Authors:** Nathan Mubiru, Rogers Mukasa, Isaac Sekitoleko, Priscilla A. Balungi, Ronald M. Kakumba, Terry Ongaria, Hubert Nkabura, Moffat Nyirenda, Anxious J. Niwaha, Wisdom P. Nakanga

**Affiliations:** 1 Clinical Diagnostic Laboratory Medical Research Council/ Uganda Virus Research Institute and LSHTM Uganda Research Unit, Entebbe, Uganda; 2 Non-communicable diseases Theme, Medical Research Council/Uganda Virus Research Institute and LSHTM Uganda Research Unit, Entebbe, Uganda; 3 Institute of Biomedical and Clinical Science, College of Medicine and Health, University of Exeter Medical School, Exeter, United Kingdom; Kwame Nkrumah University of Science and Technology, GHANA

## Abstract

**Introduction:**

Insulin and C-peptide assessment are important in characterization and management of diabetes. However, their adoption and increased clinical use in low resource settings (LRSs) is partly hindered by logistical factors including supplies required for pre-analytical sample handling and limited infrastructure. We aimed to determine the effects of altered sample processing conditions on stability of insulin and C-peptide at the pre-analytical stage.

**Methods:**

Random (non-fasted) blood samples were drawn from ten healthy (glucose range 4.1–8.0 mmol/L) participants. We investigated the mean % change from baseline of C-peptide and insulin in serum (plain tubes) vs plasma (K2EDTA tubes), centrifuged vs uncentrifuged, room vs cool box temperature on the stability of C-peptide and insulin up to 24 hours using Wilcoxon signed rank test. Results were considered clinically significant if the percentage change was > 10% and if the p-value was < 0.05.

**Results:**

Eight patients were included in the final analysis, while two were excluded due to hemolysis. C-peptide and insulin levels stayed above 90% of the baseline concentration in K2EDTA tubes across all storage and processing conditions for up to 24 hours. In contrast, samples collected in plain serum tubes kept at room temperature and uncentrifuged, C-peptide and insulin levels decreased significantly to 51% (p = 0.006) and 62% (p = 0. 083) respectively, similarly insulin levels for centrifuged samples declined to 64% (p = 0.083). All iced and centrifuged serum samples remained above 90% of baseline concentration.

**Conclusions:**

In resource-limited settings where insulin and C-peptide tests are limited to central laboratories and highly dependent on sample referral systems, these tests can be reliably measured without the need for immediate centrifugation or processing from samples collected in whole blood K2EDTA tubes uncentrifuged kept at room temperature and processed within 24hours.

## Introduction

Currently approximately 422 million people worldwide have diabetes and the majority live in low-and middle-income countries [1–3]. Appropriate diagnosis and monitoring of diabetes in people living in low-and middle-income countries is essential and can help improve the quality of life of people with diabetes [4]. Evaluation of endogenous insulin secretion, particularly outside the honeymoon period may improve diabetes management in cases where decisions regarding treatment with insulin injections have to be made [5]. This can be done by measuring circulating insulin in peripheral blood or C-peptide – a byproduct of proinsulin co-secreted in equal quantities as insulin [5–8].

Of the two, C-peptide is preferred because unlike insulin which undergoes significant hepatic extraction, C-peptide does not undergo first- pass metabolism [9,10]. Recent advances in test methods have made testing of insulin secretion using C‐peptide less expensive, more reliable and widely available even in low resource settings. Though the clinical utility of C-peptide and insulin measurement is established, their clinical use is limited by the fragility of both C-peptide and Insulin. These two molecules especially the latter are rapidly degraded both in-vivo and in-vitro by peptidase activity in the blood [11,12]. As a result, the manufacturers of these assays recommend complex pre-analytical sample handling [13,14]. The precarious requirements in the pre-analytical phase may potentially limit its utility particularly in low resource settings were such tests are limited to central laboratories. This implies that sample collection and laboratory analysis happens in different settings, most times hundreds of kilometers apart [15]. The standard pre-analytical recommendations involving immediate centrifugation and refrigeration [16] may not be feasible in the periphery settings or diabetes clinics [17]. In the field of clinical diagnostics, the stability of analytes during the pre-analytical phase is of paramount importance [18]. However, in resource-limited settings where access to sophisticated laboratory equipments and infrastructure is limited, preserving the integrity of blood samples becomes a critical concern [19]. McDonald et al. [20], Déchelotte et al. [21], and other researchers have previously shown that C-peptide and insulin are stable in dipotassium ethylenediaminetetraacetic acid (K2-EDTA) whole blood tubes at room temperature for up to 24 hours [20,21]. They also reported on insulin and C-peptide stability in samples maintained under cold chain, using highly controlled refrigeration systems maintained at 2–8°C. However, it is unclear as to whether these findings are applicable in the tropical areas of Sub-Saharan Africa (SSA) were cool boxes with ice packs are used to maintain cold chain.

We aimed to determine the most appropriate sample collection and processing criteria for insulin and C-peptide applicable in resource-limited settings by investigating stability for up to 24hours at variable conditions.

## Methods

### Ethical clearance

We conducted this study at the Clinical Diagnostics Laboratories within the Medical Research Council/Uganda Virus Research Institute and London School of Hygiene & Tropical Medicine Uganda Research Unit. Written informed consent was obtained from all participants before participating in the study. The study was approved by the Uganda Virus Research Institute Research Ethics Committee (ref: GC/127/19/03/700), Uganda National Council for Science and Technology (ref: HS 2566), and London School of Hygiene and Tropical Medicine Ethics Committee (ref: 17514). We received our final ethical approvals form UNCST on 23, April,2019 and recruited our first participant on 16, September,2019 and the last participant on 26, September 2019.

### Procedures

#### Subjects: Eligibility criteria.

Participants willing to provide informed consent, aged ≥18 years, with no history of diabetes.

After obtaining informed consent, we collected data from participants on; age, sex and fasting status using a structured questionnaire.

**Fig 1:**
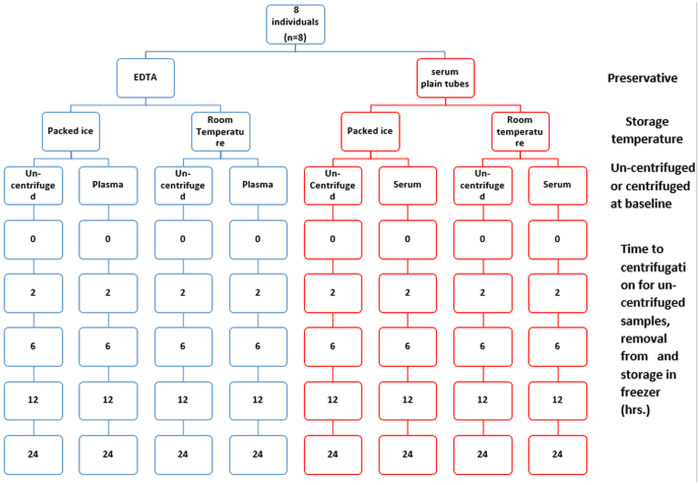
Flow diagram showing sample collection study protocol for stability of C-peptide and insulin in K2EDTA and plain serum tubes over 24 hours. At each time point samples were centrifuged at 3000 rpm for 10 minutes and supernatant stored at -20^o^C.

Non-fasting 40mls whole blood venous samples were collected for insulin and C-peptide measurements using an 18G butterfly needle and 20 ml syringe. The blood was quickly dispensed into 20 dipotassium ethylenediamineteraacetic acid (K2EDTA) tubes and 20 plain serum tubes (BD Vacutainer® tubes), Which were mixed gently by inverting (5–6) times for plain serum tubes and (8–10) times for K2EDTA tubes (Fig 1).

At zero time (baseline), four K2EDTA and four plain serum tubes were centrifuged at speed of 3000RPM for 10 minutes, for serum samples these were left to clot 30 minutes after collection prior to centrifugation. Each tube was then divided into 4 aliquots and stored in a −20°C freezer as reference specimens.

At 2, 6, 12, and 24 hours, two K2EDTA and two plain serum tubes were centrifuged at speed of 3000RPM for 10 minutes. Aliquots were prepared and divided. Two aliquots from each tube type were kept at room temperature, while the others were stored in a cool box with ice.

At each time point, one aliquot from each tube stored at room temperature and in the cool box was placed in the −20°C freezer. Additionally, four K2EDTA and four plain serum tubes were stored at room temperature. At 2, 6, 12, and 24 hours after sample collection, one tube of each type was centrifuged, aliquoted, and placed in the −20°C freezer.

Two K2EDTA and two plain tubes were stored in the cool box following the same procedure as those stored at room temperature. Ambient room temperature and cool box temperature were monitored using the calibrated Temp-Chex®, Red Spirit with measuring ranges of 15–50°C for room temperature and −5–15°C for cool box temperatures. These different temperatures were measured at time intervals of 0 min,2hr,6hr,12hr,24hr and recorded on a study temperature data collection form.

All samples were then frozen at -20^o^C for 2 weeks, then transferred to -80^o^C for 3months all frozen samples were thawed once for 30–60 minutes to reach room temperature maintained at 18–26°C. These were analyzed as a single batch at the MRC/UVRI and LSHTM Clinical Diagnostics Laboratory in Entebbe, Uganda. The Cobas 601 analyzer (Roche/Hitachi, Tokyo, Japan) was used for C-Peptide and insulin measurement using the electrochemiluminescence method. The analytical variation (CVa) of the assays during analysis of the tests for intra and inter assay precision was; CV (1.7%, 2.2%) for C-peptide and (1.5%, 3.9%) for insulin respectively which were within the manufactures acceptable limits. The (CVa) reported is from in house data following the Improvement Amendments (CLIA) Verification of Performance Specifications guiding protocol [22]. The C-peptide assay uses a direct electrochemiluminescence immunoassay using mouse monoclonal anti-C-peptide antibody labelled with ruthenium and a second mouse monoclonal anti-C-peptide antibody coupled to paramagnetic particles. The insulin assay uses a similar method of two mouse monoclonal anti-insulin antibody coupled to paramagnetic particles. For assay traceability, the insulin method has been standardized using the 1st IRP WHO Reference Standard 66/304 for insulin. The C-peptide assay has been standardized using the WHO International Reference Reagent for C-peptide of human insulin for immunoassay (IRR, code 84/510, established in 1986) from the National Institute for Biological Standards and Control (NIBSC).

We compared the results of C-peptide and Insulin samples kept in four different pre-analytic conditions: (1) un-centrifuged kept at room temperature, (2) un-centrifuged kept in a cool box with ice, (3) centrifuged kept at room temperature, (4) centrifuged kept in a cool box with ice. We assessed stability of insulin and C-peptide at the four different conditions mentioned above at 0hr,2hr,6hr,12hr and 24hr.

### Statistical analysis

Participants characteristics were summarized using frequencies and proportions for categorical variables and median interquartile range (IQR) for the continuous variables. We then computed the mean % change in C-peptide and insulin results from baseline ± 95% confidence interval. Differences between the baseline and the concentration of C-peptide and insulin at 24 hours under the 8 different pre-analytical conditions were examined using Wilcoxon signed rank since data was not normally distributed and hence opting for nonparametric tests. Our results were considered clinically significant if the percentage changes of insulin and C-peptide from baseline concentrations was > 10% [23] and statistically significant if the p value was < 0.05. Although the Clinical Laboratory Improvement Amendments (CLIA) specify Total Allowable Error thresholds of 20% for insulin and 30% for C-peptide, we used a more stringent acceptance criteria of 10% to enhance analytical reliability. We used Stata version18 to analyse the data for the P-values and Python version 3.9.12 to draw the graphs illustrating % change from baseline.


$$\begin{array}{c} \%\text{ Change}=\frac{\;new\;concentration}{baseline\;concentration\;}\;\times 100\; \end{array}$$

$$\begin{array}{c} \text{PD}=\frac{new\;concentration\;at\;time\;piont\;x-baseline\;concentration\;}{baseline\;concentration\;}\times 100\; \end{array}$$

## Results

10 participants were recruited for the primary study (eight men and two women, mean age, 30.8 ± 9) with no history of diabetes (glucose range 4.1–8.0 mmol/L). Data for 8 participants was used in the final analysis. The remaining 2 participants had heamolysed samples and where therefore excluded. The mean ambient room temperature ranged from 25–28°C and cool box temperatures ranged from 6–15°C. Internal quality controls (IQC) were documented and these all passed within the 2SD acceptable range set by the laboratory lot numbers for the PreciControl Multimarker (PCMM) were; High:36138000, Low:36138100. There was no significant difference in the mean baseline insulin levels (864 pmol/L for K2EDTA and 800 pmol/L for serum, P = 0.383) or in the mean C-peptide levels (17.3 μU/mL for K2EDTA and 14.7 μU/mL for serum, P = 0.312).

### C-peptide and insulin were more stable in samples collected and kept at room temperature in K2EDTA than those collected in plain serum tubes with or without centrifugation

**Fig 2:**
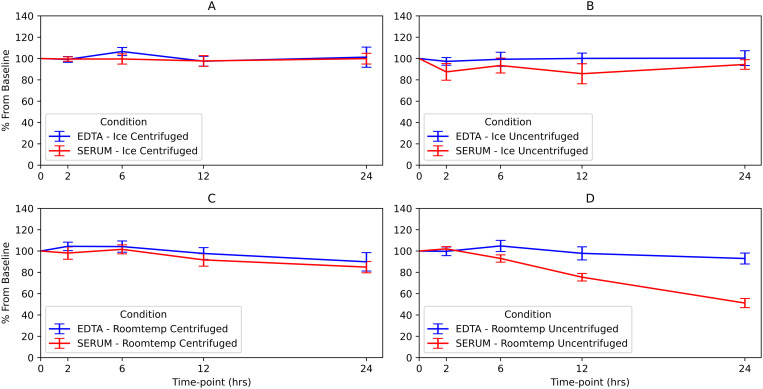
Graphical presentation of % decrease for C-peptide from baseline at different conditions.

**Fig 3:**
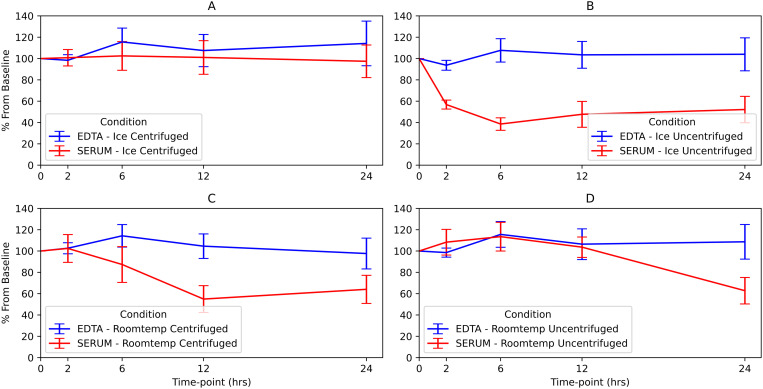
Graphical presentation of % decrease for insulin from baseline at different conditions.

C-peptide and insulin stayed more stable in unprocessed whole blood samples collected in tubes with K2EDTA compared to plain serum tubes. Over 24 hours, their levels in K2EDTA tubes remained above 90% of the initial levels (90% of baseline for C-peptide and 98% for insulin), with no significant decrease (p = 1.000 for C-peptide and p = 0.404 for insulin). In contrast, C-peptide and insulin levels significantly dropped in plain serum tubes within 24 hours, falling to 51% and 64% of baseline respectively (p < 0.001 for both). (Figs 2C, 2D, 3C and 3D).

For centrifuged samples, C-peptide in plain serum tubes dropped to 85% (p < 0.001), while insulin dropped to 62% (p = 0.083) (Figs 2D and 3D).

### C-peptide was stable in K2EDTA tubes and Serum tubes both as centrifuged and uncentrifuged kept in cooler box

C-peptide levels, in both centrifuged and unprocessed whole blood, did not decrease below 90% of baseline (p-1.00) when the samples were kept in a cooler box with ice up to 24 hours (Fig 2A and 2B).

### Insulin levels in Centrifuged and un-centrifuged K2EDTA were stable for up to 24hours when kept in a cool box

Insulin levels did not drop below 90% of baseline over 24 hours for samples kept in a cooler box with ice, both as centrifuged and unprocessed whole blood _K2EDTA_ (113% of baseline, p = 1.000 and 104%, p = 0.404 of baseline) (Fig 3A and 3).

### Insulin levels in serum remained stable when samples were centrifuged, and vice versa for un-centrifuged for samples kept in a cool box

In contrast, insulin concentrations decreased significantly in samples stored in plain serum tubes for uncentrifuged but not for centrifuged samples (52% of baseline, p = 0.006 and 97% of baseline, p = 1.000 at 24 hours respectively) (Fig 3C and 3D).

### Insulin and C-peptide levels were stable at 0 min,2hr, 6hr and 12hr in both plain serum tubes and K2EDTA for most conditions except at highlighted conditions below

C-peptide and insulin samples collected in plain serum tubes, when kept on ice and uncentrifuged, dropped below 90% of baseline within 2 hours with insulin levels dropping to 50% and C-peptide to 86% of their initial concentrations across all time points (6 and 12 hours) up to 24 hours, (Figs 2B and 3B).

C-peptide samples collected in plain serum tubes and left at room temperature without centrifugation decreased to 54% of baseline concentration by 12 hour), (Fig 2D).

### Overall stability of insulin and C-peptide in serum and K2EDTA

K2EDTA tubes improved stability and did not drop below 90% of baseline concentration across all conditions for samples collected aimed at testing insulin and C-peptide as compared to the plain serum tubes.

## Discussion

Our study found that K2EDTA performs better than plain serum tubes in maintaining stability of insulin and C-peptide. Insulin and C-peptide remain stable when collected in K2EDTA tubes for up to 24 hours, when kept at ambient room temperatures or in a cool box regardless of centrifugation. In plain serum tubes, centrifuged samples remained stable in a cool box for up to 24 hours, while uncentrifuged serum samples were stable for only 2 hours. Serum samples left unprocessed at room temperature dropped below 90% of baseline concentration by 24 hours. Overall, K2EDTA tubes improved stability of insulin and C-peptide maintaining over 90% of baseline concentration across all conditions as compared to the plain serum.

Studies by McDonald et al. [20], and Déchelotte et al. [21], support these findings showing that insulin and C-peptide are stable in K2EDTA tubes for up to 24hrs. Although Nkuna et al. [11], reported stability of up to 12hrs for C-peptide and 48hrs for insulin when collected in K2EDTA tubes left at room temperature these as well support our findings at 12hr and 24hr respectively. In contrast some assay manufacturer reported stability of 4 hours at 20–25°C for insulin and C-peptide contrary to our findings [13,14]. Previous studies documented that insulin and C-peptide samples when centrifuged immediately and kept in a cool box, were all stable regardless of whether they were collected in serum or K2EDTA tubes and are in line with ours finding, those from other authors and assay manufactures who reported stability of up to 2days at 2–8°C. [13,14,24,25]. The observed stability is likely attributed to several factors: the cool temperature, which slow down enzymatic activity; the centrifugation, which separates red cells from analytes and prevents the release of proteases due to cell lysis; and the use of K2EDTA tubes for which the anticoagulant chelates co-factors necessary for proteolytic activity.

This study has several strengths. Most notably, it evaluated the impact of delayed centrifugation a highly relevant factor for referral samples in resource-limited settings, where collection sites are often located hundreds of kilometers from testing facilities. We assed stability over 24hrs at various storage temperatures, used a cool box with ice packs which is a practical method for maintaining cold chain, and examined centrifuging time in relation to the possible times it would take referral samples to reach the testing central facility/referral laboratories.

Limitations for this study include the absence of samples with low insulin or C-peptide levels, therefore we did not evaluate how such samples would perform in this experiment and samples were tested on only one analytical platform hence where unable to compare the level of agreement between analytical platforms. Lastly, the effect of interfering factors like heamolysis and pharmaceutical compounds was not determined.

The fact that insulin and C-peptide were stable at room temperature for up to 24hrs in unprocessed K2EDTA suggests that in resource-limited settings with limited access to electricity and pre-analytical processes, samples for testing C-peptide and insulin can be collected in K2EDTA whole blood tubes and processed after at least 24hours without significant alteration. This finding simplifies sample collection procedures, especially in resource-limited settings hence the same sample can be used for HbA1c and haemoglobin concentration measurements [26,27] then centrifuged, aliquoted and tested or stored within 24hrs. Combining these results with our previous findings showing glucose stability in K2EDTA tubes, it appears that the same tube can be used for collecting samples for insulin, C-peptide and glucose testing as long as processing occurs within 6 hours and kept in a cool box [28,29].

Adoption of hand held centrifuges could provide solutions to the common challenges associated with advocating for immediate blood separation, especially in remote areas where conventional centrifuges and electricity are not readily available [28,30]. Additionally, establishment of point of care instruments that can test for insulin and C-peptide could eliminate the need for expensive pre-analytical activities involved in the sample preparation for these assays [31].

## Conclusion

In conclusion, this study has shown that insulin and C-peptide are stable in K2EDTA whole blood, without processing when kept at room temperature within 24 hours. This implies that in resource-limited settings where insulin and C-peptide tests are limited to central laboratories and highly dependent on sample referral systems, these tests can be reliably measured without the need for immediate processing provided they are tested or processed within 24hours.

## Supporting information

S1 File Table showing % percentage deviation and changes in absolute values from baseline at different time intervals up to 24hrs. (XLSX)

S2 File Dataset. (XLSX)

S3 File Field protocol: pre-analytical sample handling of venous blood for glucose, insulin and C-peptide measurement in Sub Saharan Africa: a validation exercise. (PDF)
