# Supplementary material for: EDTA tubes are suitable for insulin and C-peptide measurement in resource-limited settings and can be stored at room temperature for up to 24 hours
Source: PLoS One. 2025 Jun 30;20(6):e0312065. doi: 10.1371/journal.pone.0312065 (PMC12208407; doi:10.1371/journal.pone.0312065)
Supplement: S3 File — (PDF) [file pone.0312065.s003.pdf]

Mar 27, 2025

Pre-analytical sample handling of venous blood for glucose, insulin and C-peptide measurement in Sub Saharan Africa: A Validation Exercise

DOI
dx.doi.org/10.17504/protocols.io.5jyl8erm6l2w/v1

Wisdom Nakanga[1], Mubiru Nathan[2], Rogers Mukasa[2], Priscilla A Balungi[3], Anxious Niwaha[2], Moffat Nyirenda[2]

[1]Institute of Biomedical and Clinical Science, College of Medicine and Health, University of Exeter Medical School, Exeter, UK: Non-communicable diseases Theme, Medical Research Council/Uganda Virus Research Institute and LSHTM Uganda Research Unit, Entebbe, Uganda;
[2]Non-communicable diseases Theme, Medical Research Council/Uganda Virus Research Institute and LSHTM Uganda Research Unit, Entebbe, Uganda;
[3]1. Clinical Diagnostic Laboratory Medical Research Council/ Uganda Virus Research Institute and LSHTM Uganda Research Unit, Entebbe, Uganda

Manuscript protocols

Mubiru Nathan
MRC,UVRI,UKRI & LSHTM

OPEN ACCESS

DOI: dx.doi.org/10.17504/protocols.io.5jyl8erm6l2w/v1

Protocol Citation: Wisdom Nakanga, Mubiru Nathan, Rogers Mukasa, Priscilla A Balungi, Anxious Niwaha, Moffat Nyirenda 2025. Pre-analytical sample handling of venous blood for glucose, insulin and C-peptide measurement in Sub Saharan Africa: A Validation Exercise. **protocols.io** https://dx.doi.org/10.17504/protocols.io.5jyl8erm6l2w/v1

License: This is an open access protocol distributed under the terms of the **Creative Commons Attribution License**, which permits unrestricted use, distribution, and reproduction in any medium, provided the original author and source are credited

Protocol status: Working
We use this protocol and it's working

Created: March 06, 2025

Last Modified: March 27, 2025

Protocol Integer ID: 124476

Keywords: GPV Exercise, Cannula insertion, Blood draw , Preanalytical sample handling

Abstract

This protocol describes pre-analytical sample handling of venous blood for glucose, insulin and C-peptide measurement in Sub Saharan Africa: a validation exercise.

Guidelines

Introduction

This is a validation exercise to determining the optimal (best) preanalytical sample handling circumstance of venous blood for glucose, insulin and C-peptide measurements to ensure dependable and accurate results. We will do this by analysing the effect of time to centrifugation and storage temperature on the stability of glucose, insulin and C-peptide and secondly comparing the stability of glucose in Sodium Fluoride (NaF), Ethylenediamine tetraacetic acid (EDTA), and Serum collecting tubes while for insulin and C-peptide this will be evaluated in ETDA and serum tubes.

To do this, we shall obtain fasting blood samples from 10 willing participants who are employees of MRC UVRI LSHTM Uganda. The blood collected will be stored on either packed ice or room temperature and collected and different time points (0min,2hrs, 6hrs, 12hrs, and 24hrs) for centrifugation and storage in -20°C freezers temporally and then transfer to -80°C freezers prior to batch testing on the Cobas 6000 analyzer.

Materials

Equipment for venepuncture

- Sharps box
- Clinical waste bag
- Disposable gloves
- Paper towels
- Tourniquet
- Butterfly needles
- Tape to secure needle
- Plaster strips
- 20 or 10 ml syringes (total 60mls per participant)
- Cotton wool
- Cold box with packed ice packs
- Cold box without ice
- 4 test tube racks
- 20 Grey (NaF) tubes 2mls
- 20 Purple (EDTA) tubes 2mls
- 20 Red (Serum) tubes 2mls

Before start

This validation exercise will look at Preanalytical sample handling of venous blood, how to ensure glucose, C-peptide and insulin measurements are accurate and reliable (1)

Eligibility and consent

1 Gather all the participants together and go over the procedures.

2 Members of staff who are willing to take part in the exercise.

Bleeding preparation

3 Ask if the participants have eaten anything on the day of the bleeding.

4 Prepare the following equipment prior to arrival of the participants.

4.1 Forms and paperwork

- Field register
- Specimen forms
- Referral slips
- SPECIMEN labels for both the test tubes and aliquots.

4.2 Equipment for venepuncture

- Sharps box
- Clinical waste bag
- Disposable gloves
- Paper towels
- Tourniquet
- Butterfly needles
- Tape to secure needle
- Plaster strips
- 20 or 10 ml syringes (total 60mls per participant)
- Vacutainers per participant:
- ♣ 20 Grey (NaF) tubes 2mls
  ♣ 20 Purple (EDTA) tubes 2mls
  ♣ 20 Red (Serum) tubes 2mls
- Cotton wool
- Cold box with packed ice packs
- Cold box without ice
- 4 test tube racks

Preparation of the test tubes

5 Prepare two test tube racks the first one filled with 20 grey, 20 purple and 20 red test tubes and the other with 60 aliquot tubes.

PRIMARY BLOOD COLLECTING TUBES

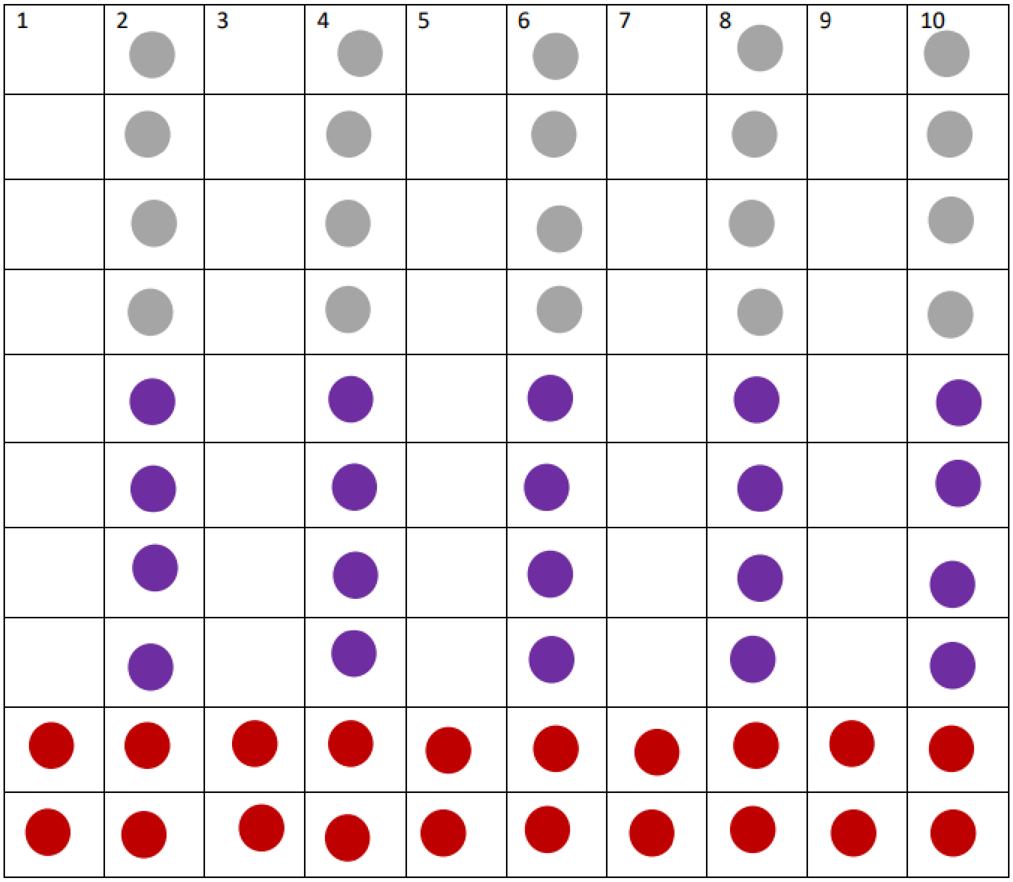

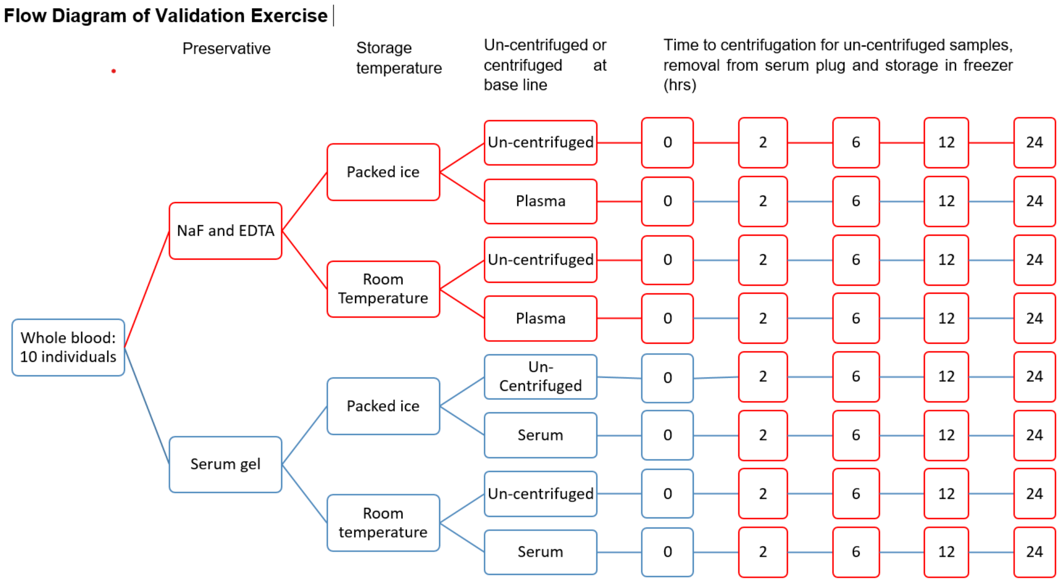

Figure 1Flow diagram detailing sample
collection protocol for the study over 24 hrs. At each time point the samples will be centrifuged for 10 minutes. The supernatant will be frozen at -20^{0}C

6 Paste the prepared barcode identifier onto the test tubes and its corresponding aliquot.

7 Secure two other racks, one that will be placed on Room temperature and the other that will be placed in the cooler box with packed ice.

Cannula insertion, blood draw and pipetting into the tubes

8 Into the left arm, insert a butterfly needle using aseptic non-touch technique ANTT and tape it into position.

9 From the butterfly needle, take blood into the 10 ml syringes.

10 When the syringe is full, remove the syringe, making sure that blood does not spill, and pass it on to the partner and place another syringe onto the needle until a total of 60 mL has been collected from the participant.

11 The partner pipettes 1 mL of blood into the already prepared test tubes until all 60 tubes are filled with blood.

Centrifugation and aliquot of initial samples

10m

12 Centrifuge the initial samples according to the lab request form (see below).

GLUCOSE PREANALYTICS HANDLING VALIDATION EXERCISE – LABORATORY REQUEST FORM								
Study Name	GPV EXERCISE	Study Site			Visit Date	Day	Month	Year
Participant ID			Sex	M F	DOB	Day	Month	Year
			Initial					
Sample Collection Date			Collected by (Initials)					
Sample Type	☐ 20 NaF tubes(Grey Top)<br>☐ 20 EDTA tubes(Purple Top)<br>☐ 20 Serum tubes(red top)				Sample collection time	24 hour clock		
							:	
					Requested by (initials)			

Laboratory Request				
**Time of sample processing**	**Planned time**	**Actual time**	**Action: Aliquot**	**Action: Store in -20°C**
**0min after blood collection**			☐ 12 tubes at time 1: NCU1, NCC1, NRU1, NRC1, ECU1, ECC1, ERU1, ERC1, SCU1, SCC1, SRU1, SRC1<br>☐ 8 NaF tubes: NCC2, NCC3, NCC4, NCC4, NRC2, NRC3, NRC4, NRC5<br>☐ 8 EDTA tubes: ECC2, ECC3, ECC4, ECC5, ERC2, ERC3, ERC4, ERC5<br>☐ 8 Serum tubes: SCC2, SCC3, SCC4, SCC5, SRC2, SRC3, SRC4, SRC5	☐ 12 tubes at time 1: NCU1, NCC1, NRU1, NRC1, ECU1, ECC1, ERU1, ERC1, SCU1, SCC1, SRU1, SRC1
**6hrs after blood collection**			☐ 2 NaF tubes: NCU2,NRU2<br>☐ 2EDTA tubes: ECU2, ERU2<br>☐ 2 Serum tubes: SCU2, SRU2	☐ 12 tubes at time 2: NCU2, NCC2, NRU2, NRC2, ECU2, ECC2, ERU2, ERC2, SCU2, SCC2, SRU2, SRC2
**12hrs after blood collection**			☐ 2 NaF tubes: NCU3,NRU3<br>☐ 2EDTA tubes: ECU3, ERU3<br>☐ 2 Serum tubes: SCU3, SRU3	☐ 12 tubes at time 3: NCU3, NCC3, NRU3, NRC3, ECU2, ECC3, ERU3, ERC3, SCU3, SCC3, SRU3, SRC3
**24 hrs after blood collection**			☐ 2 NaF tubes: NCU4,NRU4<br>☐ 2EDTA tubes: ECU2, ERU4<br>☐ 2 Serum tubes: SCU4, SRU4	☐ 12 tubes at time 4: NCU4, NCC4, NRU4, NRC4, ECU4, ECC4, ERU4, ERC4, SCU4, SCC4, SRU4, SRC4
**48hrs after blood collection**			☐ 2 NaF tubes: NCU5,NRU5<br>☐ 2EDTA tubes: ECU5, ERU5<br>☐ 2 Serum tubes: SCU5, SRU5	☐ 12 tubes at time 5: NCU5, NCC5, NRU5, NRC5, ECU5, ECC5, ERU5, ERC5, SCU5, SCC5, SRU5, SRC5

13 Centrifuge for 3000 rpm, 00:10:00 . 10m

Placement of test tubes in cooler box or room temperature

14 Place the test tubes and aliquots in the rack to be placed in the cooler box or to be left at Room temperature according to table 3 and 4 below.

INITIAL TEST TUBE RACK FILLED WITH NAF, EDTA AND SERUM TEST TUBES

	A	B	C	D	E	F	G	H
				Time to centrifugation for uncentrifuged samples and storage in freezer (hrs)				
				0	2	12	24	48
	NAF	Crushed Ice	Uncentrifuged	1	1	1	1	1

A	B	C	D	E	F	G	H
		Centrifuge and Aliquoted	1	1	1	1	1
	Room Temperature	Uncentrifuged	1	1	1	1	1
		Centrifuge and Aliquoted	1	1	1	1	1
EDTA	Crushed Ice	Uncentrifuged	1	1	1	1	1
		Centrifuge and Aliquoted	1	1	1	1	1
	Room Temperature	Uncentrifuged	1	1	1	1	1
		Centrifuge and Aliquoted	1	1	1	1	1
Serum	Crushed Ice	Uncentrifuged	1	1	1	1	1
		Centrifuge and Aliquoted	1	1	1	1	1
	Room Temperature	Uncentrifuged	1	1	1	1	1
		Centrifuge and Aliquoted	1	1	1	1	1

LABELING OF INITIAL TEST TUBES AND OF ALIQUOTS

A	B	C	D	E	F	G	H
			Time to centrifugation for uncentrifuged samples and storage in freezer (hrs)				
			0	6	12	24	48
NAF	Crushed Ice	Uncentrifuged	NCU 1	NCU 2	NCU 3	NCU 4	NCU 5
		Centrifuge and Aliquoted	NCC 1	NCC 2	NCC 3	NCC 4	NCC 5
	Room Temperature	Uncentrifuged	NRU 1	NRU 2	NRU 3	NRU 4	NRU 5
		Centrifuge and Aliquoted	NRC 1	NRC 2	NRC 3	NRC 4	NCR 5
EDTA	Crushed Ice	Uncentrifuged	ECU 1	ECU 2	ECU 3	ECU 4	ECU 5
		Centrifuge and Aliquoted	ECC 1	ECC 2	ECC 3	ECC 4	ECC 5
	Room Temperature	Uncentrifuged	ERU 1	ERU 2	ERU 3	ERU 4	ERU 5

A	B	C	D	E	F	G	H
		Centrifuge and Aliquoted	ERC 1	ERC 2	ERC 3	ERC 4	ERC 5
Serum	Crushed Ice	Uncentrifuged	SCU 1	SCU 2	SCU 3	SCU 4	SCU 5
		Centrifuge and Aliquoted	SCC 1	SCC 2	SCC 3	SCC 4	SCC 5
	Room Temperature	Uncentrifuged	SRU 1	SRU 2	SRU 3	SRU 4	SRU 5
		Centrifuge and Aliquoted	SRC 1	SRC 2	SRC 3	SRC 4	SRC 5

RACK TO BE PLACED IN COOLER BOX WITH TEST TUBES AND ALIQUOTS

A	B	C	D	E	F	G
		Time to centrifugation for uncentrifuged samples and storage in freezer (hrs)				
		0	6	12	24	48
NAF	Uncentrifuged	1	1	1	1	1
	Aliquot	1	1	1	1	1
EDTA	Uncentrifuged	1	1	1	1	1
	Aliquot	1	1	1	1	1
Serum	Uncentrifuged	1	1	1	1	1
	Aliquot	1	1	1	1	1

RACK TO BE PLACED AT ROOM TEMPERATURE WITH TEST TUBES AND ALIQUOTS

A	B	C	D	E	F	G
		Time to centrifugation for uncentrifuged samples and storage in freezer (hrs)				
		0	6	12	24	48
NAF	Uncentrifuged	1	1	1	1	1
	Aliquot	1	1	1	1	1
EDTA	Uncentrifuged	1	1	1	1	1
	Aliquot	1	1	1	1	1
Serum	Uncentrifuged	1	1	1	1	1
	Aliquot	1	1	1	1	1

15 At the different time points (0min, 6 hrs, 12hrs, 24hrs, 48hrs) centrifuge aliquot the initially uncentrifuged samples and store all samples in -20 °C freezers for 2weeks and in -80^{0}C prior to batch testing.

SAMPLES IN COOLER BOX

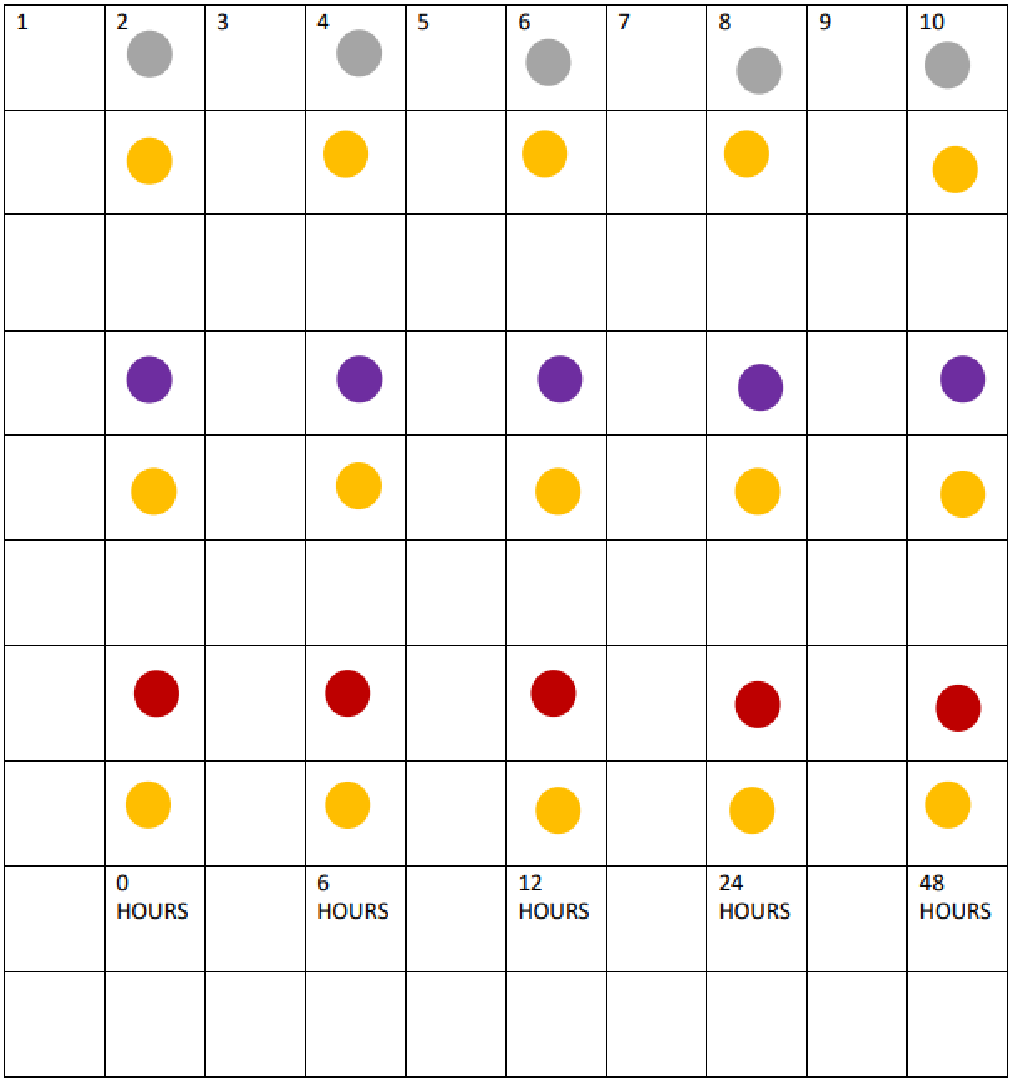

SAMPLES AT ROOM TEMPERATURE

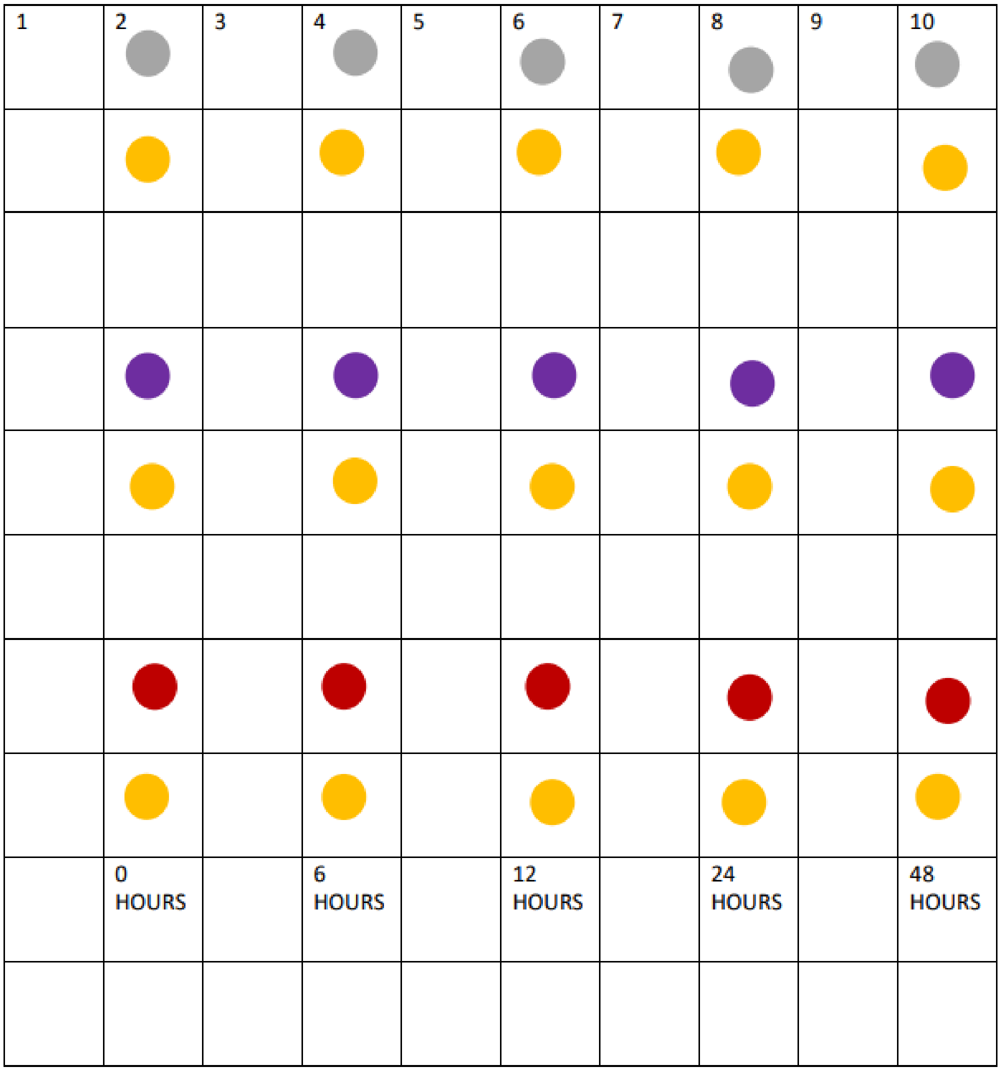

FINAL ALIQUOT RACK IN FREEZER

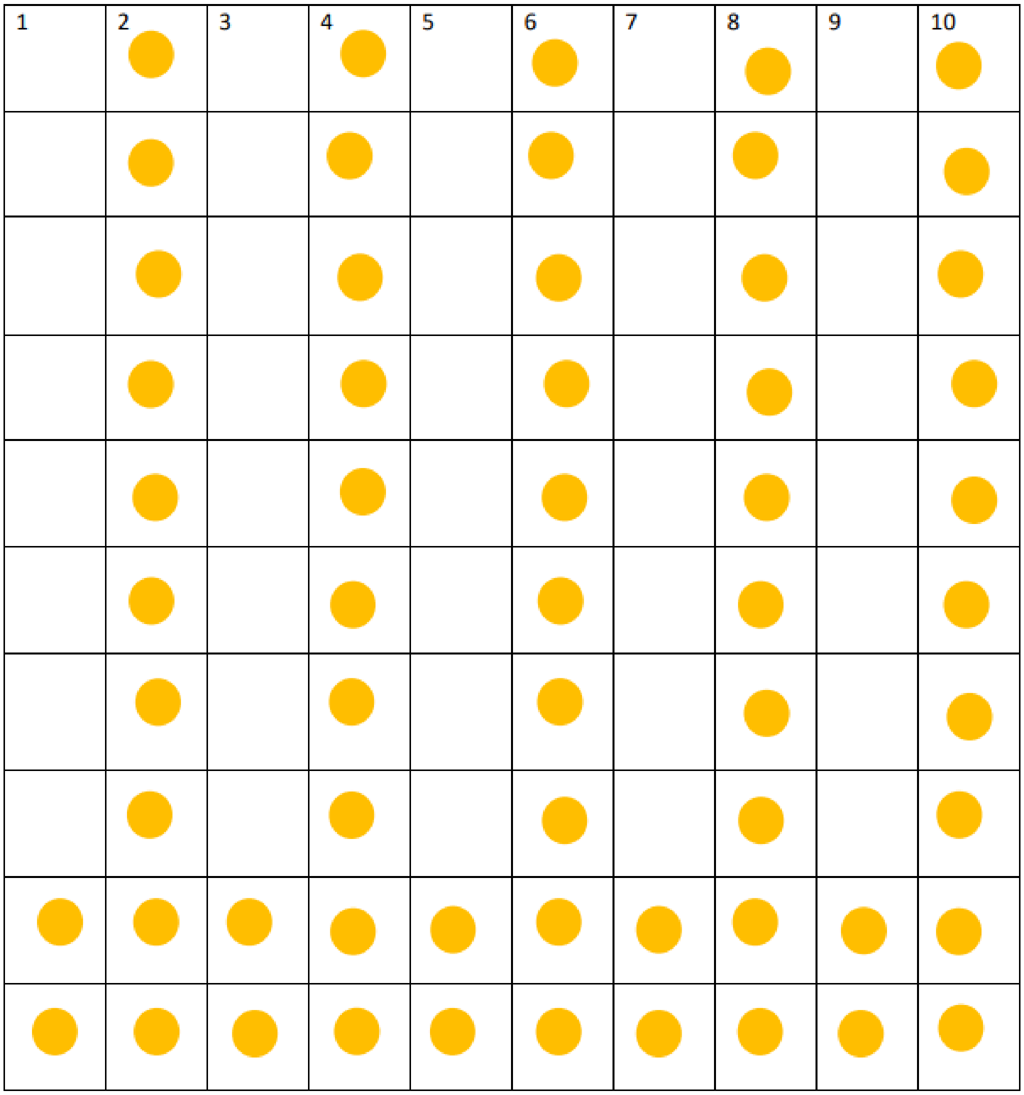

16 Record in the lab request form.

Protocol references

1. Steele, A., et al., *Preanalytical sample handling of venous blood: how to ensure your glucose measurement is accurate and reliable.* Practical Diabetes, 2013. **30**(3): p. 128-131.
